# Migrants from Ukraine in the Polish labour market as perceived by Poles from rural areas and towns

**DOI:** 10.1371/journal.pone.0306895

**Published:** 2024-09-13

**Authors:** Wioletta Knapik, Lidia Luty, Monika Zioło, Monika Odlanicka-Poczobutt

**Affiliations:** 1 Faculty of Agriculture and Economics, University of Agriculture in Krakow, Krakow, Poland; 2 Faculty of Organization and Management, Silesian University of Technology, Gliwice, Poland; Medical University of Warsaw, POLAND

## Abstract

The article is devoted to presenting the topic of migration of Ukrainian nationals to Poland. The work makes use of a survey under a project carried out in Polish rural areas and small towns. Seven hundred interviews were held in total. We conducted a quantitative analysis of its results here. The employed methods involve variable frequency distribution. The independence of the features was tested with the non-parametric chi-square test of independence. The association of the investigated variables was determined with Cramér’s V. The research shows that the most numerous foreign nationals in the Polish labour market in 2021 were Ukrainians. The positive trend started in 2017. The respondents perceived the migration of Ukrainian nationals to Poland mostly positively, especially regarding seasonal work. They also emphasized that the Ukrainians performed work at variance with their qualifications. Only every fifth participant agreed that migrants took away jobs from Poles. Most of the respondents pointed out that small business owners benefited from employing Ukrainians. The overwhelming majority of the respondents noted an increase in migration from Ukraine after the full-scale invasion and that entire families of Ukrainians were coming to Poland. Nearly half of them agreed that the support system for Ukrainian migrants was a burden on municipal budgets.

## Introduction

Migration of Ukrainian nationals to Poland should be considered in two broader contexts, economic and sociocultural [1–3]. In 2022, the media announced Poland was facing a situation nobody would expect a few months back. Over two million Ukrainian nationals fleeing the Russian aggression were crossing the Polish border [4]. Over one million of them could stay for longer.

Many as 12 million people may want to leave Ukraine permanently and that refugee policies in potential destination countries are likely to have a substantial impact on the distribution of Ukrainian refugees between different countries [5].

The Polish government had no migration policy and the country was on the brink of an unparalleled migration crisis. No refugee camps were built in Poland but the relocation mechanism seemed to be necessary. Especially with the Polish parliamentary elections in 2023. Anti-immigrant spin is only to be expected during the campaign.

Between June and August 2022 there were conducted a survey of refugees in the border provinces of Podkarpackie voivodeship and Lubelskie voivodeship to identify their actual needs and expected scope of long-term assistance and support. The aim of the survey was to obtain basic information about refugees from Ukraine who left their country across the Polish-Ukrainian border in 2022. It was particularly important to identify their health needs.

The war forced Ukrainians from across the country to flee. Most of them (72%) lived over 500 km from a Polish border crossing. Every fifth refugee lived 101 km to 500 km from a Polish border crossing. Statistics on foreign nationals in the Polish labour market were completely dominated by Ukrainians in 2021. In 2021, Ukrainian nationals were granted 325,213 work permits (64.5% of all permits for foreign nationals), which was a 68.9% increase compared to 2017. The trend reversed in 2022. The number of issued permits for Ukrainian nationals shrunk by 74%, which is 240,000 permits. The change was not due to a smaller number of employees from Ukraine but the Act on aid to Ukrainian nationals [6], which allows for employment without a permit.

The economic cooperation is growing as Ukrainian businesses move to Poland because of the war, taking advantage of the geographic proximity. Those Ukrainian companies that had already been present in 2022, expand with new branches. Ukrainian nationals become sole proprietors or find employment with Polish companies. The success of these endeavours hinges on historical and cultural factors to a large extent. The number of businesses started by Ukrainian nationals in Poland has been gradually rising since the full-scale invasion. The period from March 2022 to the end of January 2023 saw a total of 17,764 new sole proprietorships. Most of the new Ukrainian businesses recorded with the Polish Central Registration and Information on Business are situated in Mazowieckie Voivodeship (24%), Dolnośląskie Voivodeship (15%), Małopolskie Voivodeship (14%), and Pomorskie Voivodeship (11%). Ukrainian businesswomen make up 41% of all the owners. The structure of new businesses by sex does not reflect the demographic structure of war refugees. The register of Ukrainian nationals and their families who have been granted refugee status under the Act (discussed later in the article) has over 985,000 people (as on 20.02.2023), 49% of which are women and 11% are working-age men [7].

Data from the Ministry of Labour and Social Policy, Central Statistical Office, and Social Insurance Institution show that recent years saw an increase in the number of foreign nationals employed in Poland. This means Poland has reached the primary goal of its immigration policy liberalised in 2007–2008 to increase the influx of foreigners to Poland and their employment. The grounds for the liberalising Acts assumed seasonal employment of foreign nationals from five Eastern European countries would occur in those professions that Poles find unattractive. The current data neither support nor negates the premise. Still, the hypothesis that the increase in foreign workforce did not affect the employment level of Poles seems justified considering employer declarations of entrusting work to foreign nationals and sectors manned by foreigners. It is evident from the relative employment stability of Poles in sectors where foreigners are most often employed. This suggests a complementary nature of seasonal employment of foreign nationals and Poles, which means the liberalisation of the immigration policy in Poland is [8, 9].

To conduct a comprehensive analysis of labour migration from Ukraine to Poland, it is necessary to consider the employment of Ukrainians from 2010 to 2019. This segment of the Polish labour market has undergone significant changes. Although in 2010, 52% of Ukrainians in the Polish labour market worked in agriculture, mainly fruit-picking seasonal jobs, 23% in the construction industry, 21% in services, and merely 4% in industry, in 2019, 60% worked in services and only 12% in agriculture (14% in industry and construction each) [10].

A new type of seasonal work permit issued by district labour offices on behalf of District Starost was introduced on 1 January 2018 [11]. The number of seasonal work declarations issued to Ukrainian nationals gradually increased from 2013 to 2017. It declined by 15.67 pp. year-on-year in 2018. The fluctuations persisted until 2020. In 2021, the number rose nearly to the all-time high of 2017. The mean share of permits for Ukrainians compared to other nationals was 93.1% from 2013 to 2020. In 2021, it was much lower, around 82.5%. In a similar manner, the Act on aid to Ukrainian nationals related to the war in Ukraine of 12 March 2022 streamlines their access to the Polish labour market. An amendment to this act signed on 26 March 2022 by the President of the Republic of Poland effective retroactively of 24 February 2022 specifies that foreign nationals not covered by the Act who are displaced persons listed in the implementing decision of the EU Council may apply for the temporary protection under the Act on protection of foreign nationals in the territory of Poland:

- by crossing the Polish border, Ukrainian nationals acquire the right to legal stay and access to the labour market in Poland for 18 months. Should the circumstances so demand, the period can be extended by another 18 months, totalling three years;
- Ukrainian nationals can obtain a PESEL identification number, which streamlines official contacts, such as medical treatment and access to electronic medical documents;
- the refugees have a right to family benefits, parenting benefits, Dobry Start school benefit, family care capital for every child aged 12 to 35 months, subsidy for daycare centre, children’s club, or daycare provider, and welfare benefits under general regulations;
- Ukrainian nationals can start and conduct business in Poland just as Polish nationals. The prerequisite is to have a PESEL number [12].

The growth of the number of Polish population driven by migration has exacerbated problems that Poland faced even before the Russo-Ukrainian war [13, 14]. Long-term integration of Ukrainians will require substantial expenses, while public engagement and the general spirit from the early days of the full-scale invasion are abating.

In addition, the anti-democratic policies of the Polish government, especially in the last months of 2023, have contributed to building an anti-Ukrainian sentiment [15].

The European Commission provided EUR 348 million for various aid programmes from 28 February 2022 (of the declared EUR 500 million). A humanitarian fund of EUR 11.6 billion is planned for 2021–2027. It is spent on food, water, basic household products, healthcare, psychosocial support, emergency shelters, and the basic needs of those in the worst situation. The humanitarian aid in Ukraine reached eleven million people as of today. Over 8.9 million people were given food, 4.4 million received medical interventions or supplies, and over 2 million, cash. Note that even before the invasion, the EU granted Ukraine various support loans amounting to EUR 5 billion from 2014 to 2021. After the invasion, the European Commission proposed a new EUR 1 billion macro-financial assistance operation for Ukraine in the form of long-term loans on favourable terms on 1 July 2022. It is the first part of the exceptional macro-financial assistance package of up to EUR 9 billion announced in the Commission’s communication on assistance to and reconstruction of Ukraine of 18 May 2022 and endorsed by the European Council on 23–24 June 2022 [16].

In March 2022, the EU triggered the Temporary Protection Directive, an EU emergency response system applied to mass influxes of migrants. The directive provides for:

- immediate collective protection for displaced persons;
- relieving national asylum systems in EU countries.

The rights of the beneficiaries of temporary protection include a residence permit, access to employment, access to accommodation or housing, access to medical care, and access to education for minors [17].

The Polish Act on aid to Ukrainian refugees provides for principles of regularising stay and benefits and discounts for Ukrainian nationals. Poles who host refugees from Ukraine will also receive compensation benefits. Institutions and individuals who provided accommodation and food to Ukrainian nationals who arrived in Poland from Ukraine due to the war, as well as Ukrainian nationals holding a Pole’s Card who arrived with their immediate family, will be eligible for a cash benefit. The Act was amended on April 30 to extend the benefit provision for up to 120 days after the Ukrainian national’s arrival in Poland. The benefit period may be extended due to exceptional circumstances. The benefit amount in PLN 40 a day per person [18].

## Material and method

**Fig 1:**
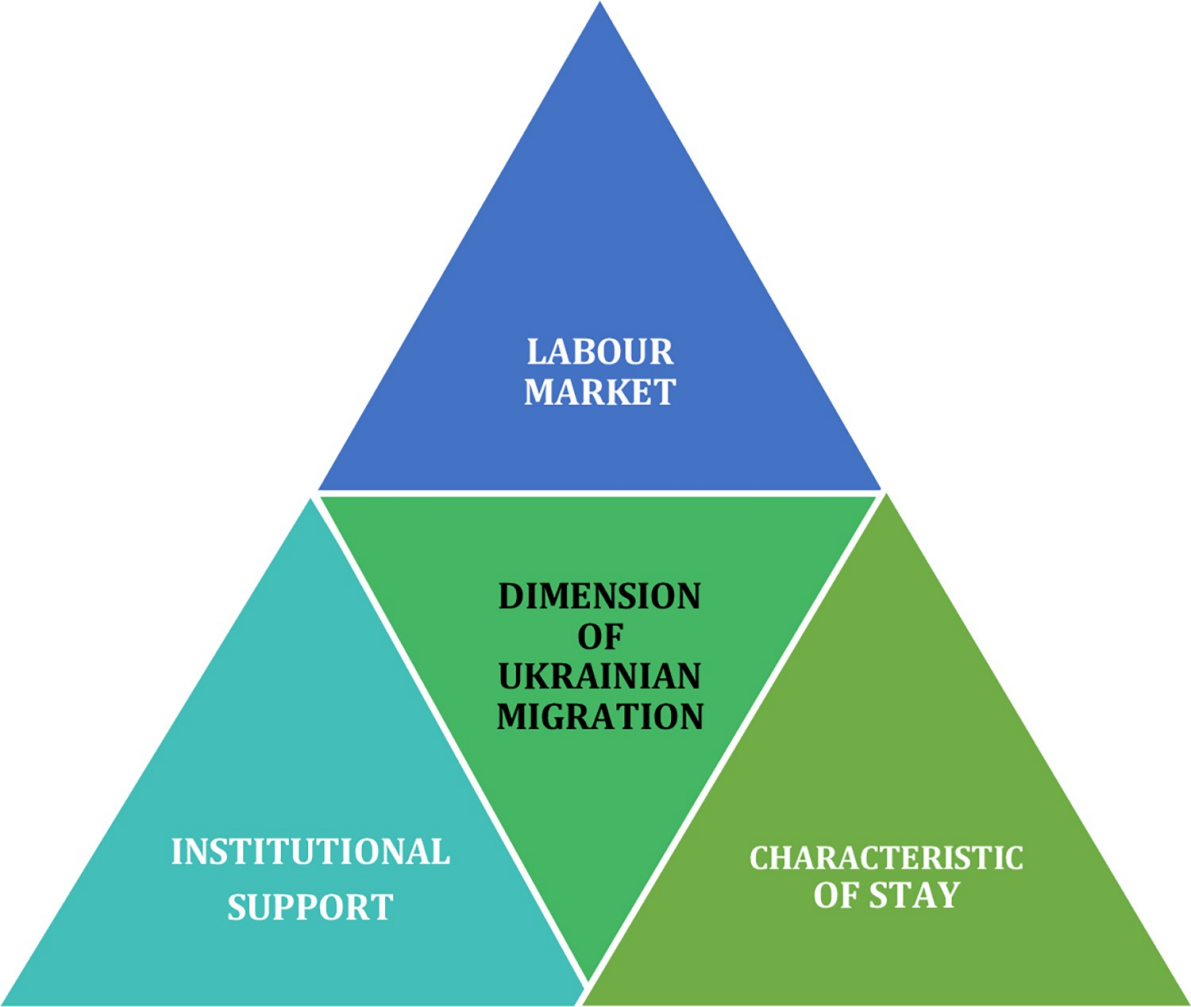
Dimensions of Ukrainian migration assessed by the respondents.

The research aims to investigate the situation of Ukrainian nationals in the seasonal labour market in rural areas and towns in Poland. The problem is presented before the full-scale Russian aggression on Ukraine and during the war against the backdrop of cultural and social conditions, mainly in the context of support for war refugees, their problems, and relationships between Poles and Ukrainians. The study is founded on surveys intended to gather information on seasonal work conditions and perception of Ukrainian immigrants. It reflects a subjective assessment of moods concerning the investigated problem among residents of rural and town areas in Poland. The research objectives are to diagnose the conditions of seasonal labour migration in three areas: 1. labour market, considering sex, age, education, and region of residence of the respondents; 2. characteristics of stay; and 3. institutional support for Ukrainian migrants (Fig 1).

In the field of the labour market, respondents were asked several questions related to their opinions on the negative impact of refugees on destabilizing the economy and taking jobs away from Poles. On the positive side, the interviewees were asked whether small enterprises benefit from additional labour force and whether Poland gains more seasonal workers. Additional questions encompassed consistency of current jobs of migrants with their qualifications and if they form long-term relations with their employers.

Next dimension which the survey encompassed was a characteristic of stay. In this aspect respondents were asked on the impact of war on the number of migrants, length of their stay and whether they settled in Poland alone or with their families.

The last aspect addressed was related to the institutional support for Ukrainian migrants. In this section, respondents were asked about various types of financial and non-financial assistance provided to migrants by the government, that they were aware of.

The employed methods involve variable frequency distribution. The independence of the features was tested with the non-parametric chi-square test of independence. The association of the investigated variables was determined with Cramér’s V. The analyses were conducted in Dell Statistica v. 13.1 (StatSoft Polska). Frequency analysis is used to represent information on the general number of occurrences and percentages of individual responses to reveal the data structure. This is how it has been employed in the article. Cramer’s V is a chi-squared measure of the degree of association between variables. The coefficient was calculated for those variables for which the chi-squared test of independence indicated a statistically significant association (at a significance level of 0.05).

Using the Likert scale popular in marketing, market, and social surveys as a model, the authors decided to measure responses on a bipolar, five-point scale. The respondents chose their answers on a scale from complete acceptance to complete rejection, with a neutral position in the middle.

**Fig 2:**
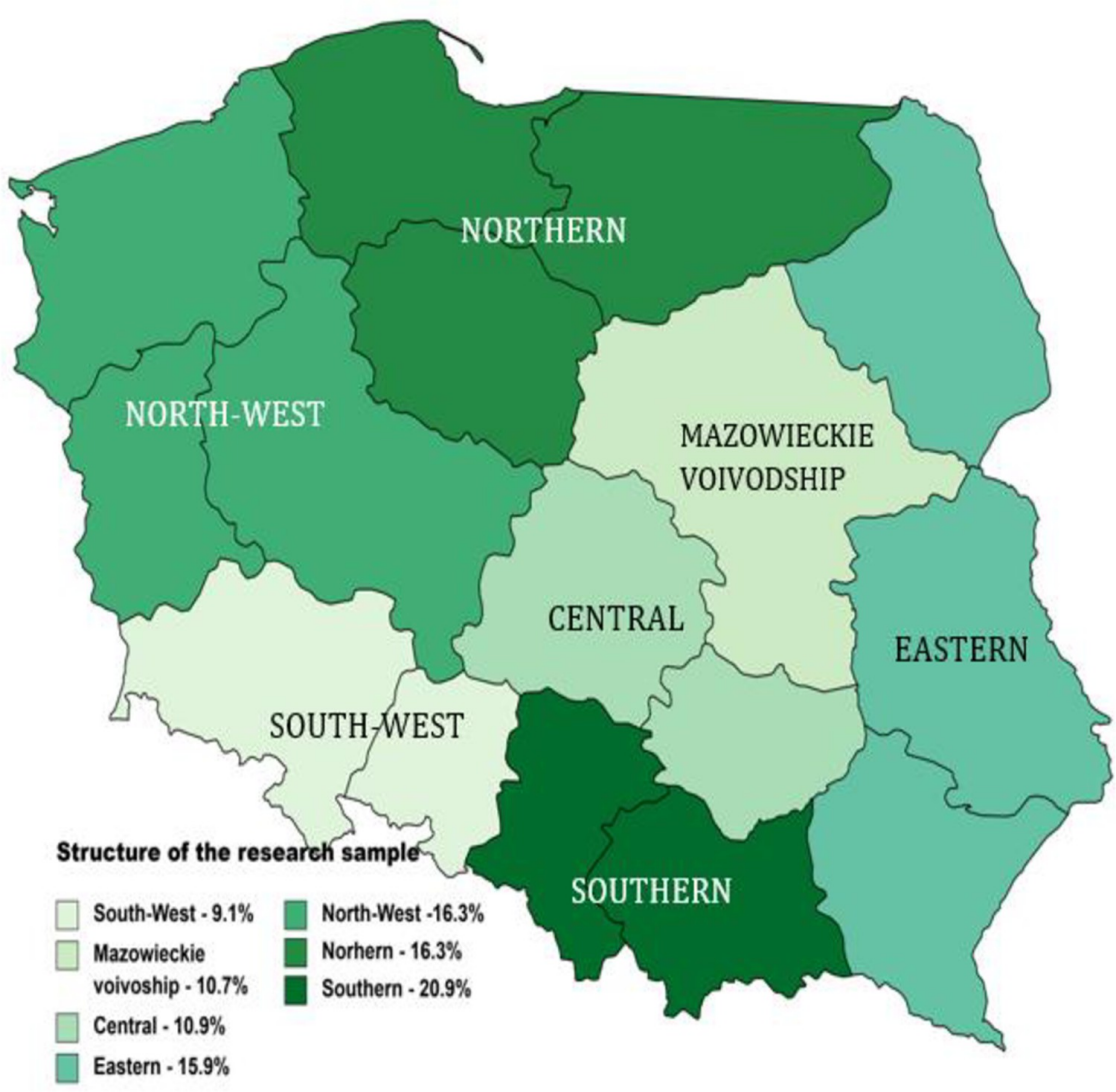
Structure of the survey sample according to macro-regions of Poland (NUTS 1). A new macro-region scheme was introduced in Poland on 1 January 2018 with an additional macro-region, Mazowieckie Voivodeship with the capital of Poland, Warsaw. It is because of the strong impact of the agglomeration on statistics of a large central region with a different profile of urban areas much smaller than the capital.

The research is part of the project ‘National Identity of Poles in Light of Migration of Ukrainian Nationals. Prevention of Social Conflicts’ was part of the programme of the Ministry of Education and Science ‘Science for the Public’. The diagnosis of economic dimensions of seasonal migrations of Ukrainian nationals into Polish rural areas and towns employed an original survey questionnaire. The survey was conducted using a computer-assisted telephone interview (CATI) method. It involved a representative sample of adult residents of rural areas and towns. The sampling frame consisted of a database of telephone numbers from rural areas and towns with populations up to fifteen thousand residents. A total of 5,000 randomly selected telephone numbers were used, with a 14% response rate. In total, seven hundred interviews were conducted. The database was purchased legally from ZN Direct sp. z o.o. Up to five attempts were made to reach each respondent. The preliminary assumption was to interview a proportional share of residents in every NUTS 1 unit (Fig 2). The actual sample was consistent with the planned structure (up to 3 pp. difference). The survey was conducted in March 2023.

Both sexes had a similar representation in the sample. The largest group of respondents was aged 35–54, which was 45.7% of the sample. Most of the respondents held a high school or university diploma (Table 1).

**Table 1:** Profile of the sample [%].

Specification			Symbol	Structure	
				Number	Percentage
	Sex	Female	f	355	50.7
		Male	m	345	49.3
	Age	18–34 years	I	111	15.8
		35–54	II	320	45.7
		55 and over	III	269	38.5
	Education	Basic^a^	(b)	133	19.0
		Secondary	(s)	287	41.0
		Higher	(h)	280	40.0
	Macro-region	North	N	114	16.3
		North-West	NW	114	16.3
		Mazowieckie Voivodeship	MV	75	10.7
		Central	C	76	10.9
		South-West	SW	64	9.1
		South	S	146	20.9
		East	E	111	15.9
**TOTAL**				700	100

^a^: primary education, vocational education, or no answer

The literature review covers research areas presented in the Results section, i.e. 1. labour market; 2. characteristics of stay; and 3. institutional support for Ukrainian migrants.

## Results

The liberalization of access to the Schengen zone for Ukraine nationals in 2017 allowed many Ukrainians to travel to the Entire European Union (except for Ireland and Great Britain), Iceland, Lichtenstein, Norway, and Switzerland visa-free. This event undoubtedly increased the popularity of Poland as the destination for Ukrainian nationals for whom geographic and cultural proximity are decisive factors. Ukrainian nationals can work without a visa only in Poland as long as they have a biometric passport and a declaration of employment. With a Polish work permit, Ukrainian employees gain protection under the Polish Labour Law. Polish employers are obliged to conform to all employment requirements, including registering Ukrainian employees with the Social Insurance Institution [19].

### 1. Labour market

Most of the respondents exhibited a positive perception of the Ukrainian migration to Poland. The majority highlighted the benefit of an additional workforce for seasonal work, a point emphasized by over 82% of the respondents. Only 20.6% of the participants agreed that migrants took away jobs from Poles. Over 60% of the interviewees pointed out that small business owners benefited from employing Ukrainians. Note that nearly 40% of the respondents believed that Ukrainians worked jobs not matching their qualifications. It may be because of the language barrier preventing migrants from taking up jobs in line with their trade, which hurts both them as they are employed beneath their qualifications and the Polish labour market as it cannot access the full potential of the new employees. Most respondents were unsure whether migrants who settled in Poland for an extended period returned to their previous employers.

**Fig 3:**
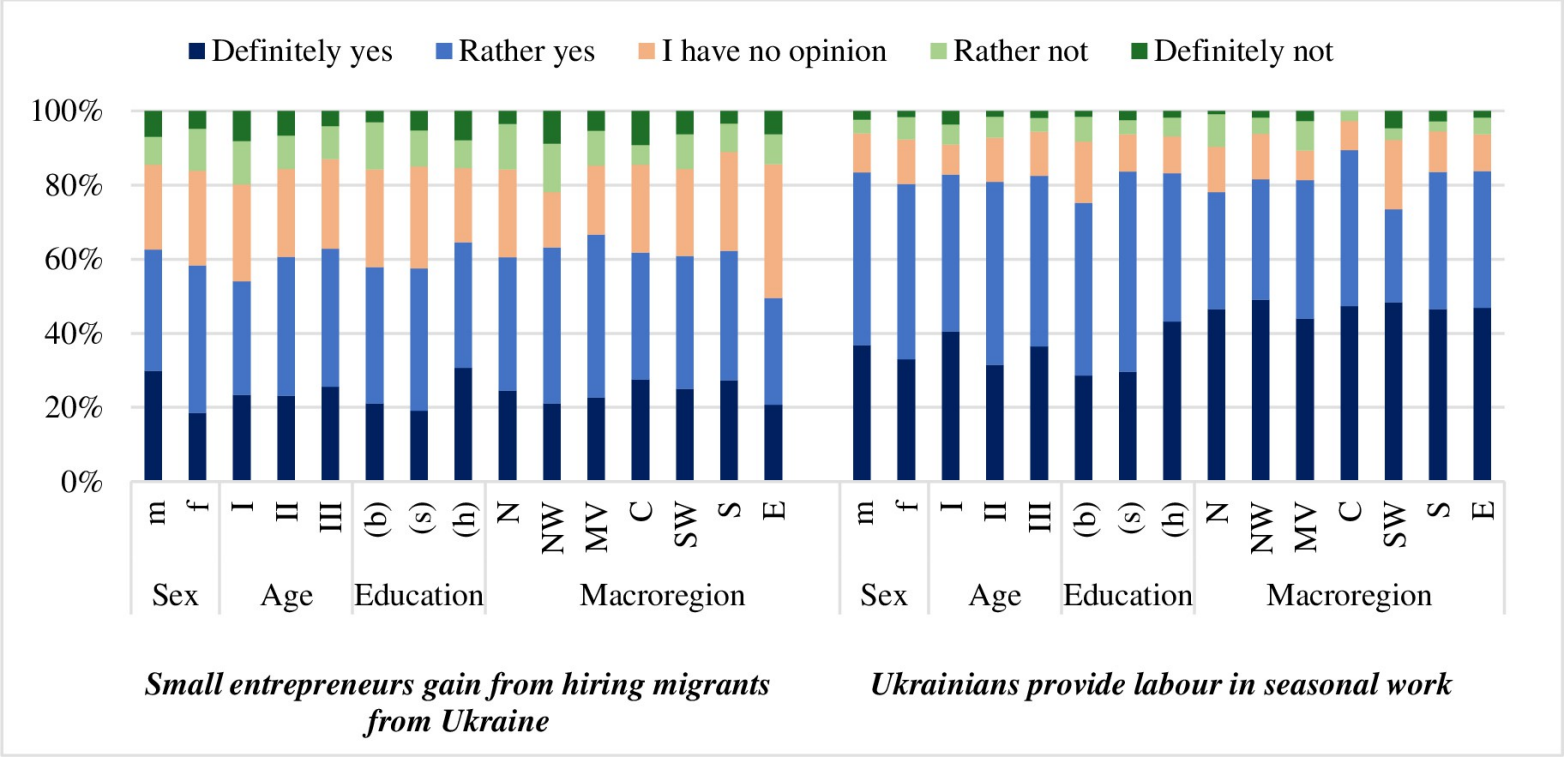
Opinions of the respondents about the impact of Ukrainian migrants on the labour market in Poland by sex, age, education, and macro-region. Symbols as per Table 1.

The opinion of Poles on Ukrainian refugees was positive in each investigated aspect. Poles pointed out the benefits of the influx of additional labour. The youngest age group was also the most sceptical about the new employees, perhaps because of the greatest risk of losing jobs in favour of refugees (Fig 3). Consequently, they more often believed that migrants from Ukraine were undercutting the economy and taking jobs away from Poles.

The educational background of the respondents is a substantial differentiating factor regarding the attitude of Poles towards Ukrainians (Table 2). People with university degrees whose jobs are not threatened so much more often pointed out that the refugee influx was beneficial for small business owners and provided a workforce for seasonal increases in labour demand. The lower the education, the greater the probability of belief that refugees had a detrimental impact on the Polish labour market.

**Table 2:** Results of the test of independence of respondents’ opinions about the impact of migrants from Ukraine on the labour market in Poland by sex, age, education, and residence.

Specification								
	Sex		Age		Education		Macro-region	
	*p-value*	Cramér’s V	*p-value*	Cramér’s V	*p-value*	Cramér’s V	*p-value*	Cramér’s V
*Small entrepreneurs gain from hiring migrants from Ukraine*								
	**	0.151	-		*	0.118	-	
*Ukrainians provide labour for seasonal work*								
	-		-		**	0.124	-	
*Ukrainians destabilise the Polish economy*								
	-		*	0.114	***	*0*.*152*	*	0.109
*Ukrainians take jobs away from Poles*								
	-		**	0.135	***	0.169	-	
*Bonds form between long-term settled labour migrants and employers*								
	-		-		-		*	0,122
*Migrants have jobs consistent with their qualifications*								
	-		-		-		-	

p value

* *p* < 0.05

** p < 0.01

*** p < 0.001

‘-’ p > 0.05

The regional background also played a role in the perception of the refugee impact on the economy. Respondents from North and Mazowieckie Voivodeship less often agreed with the notion that the refugees were destabilising the economy. The Central and East macro-regions had more affirmative responses.

Macroregion influenced also the opinion of respondents on whether refugees form long-term relationships with their employers. People from the North-Western and Central regions were particularly likely to notice this phenomenon. Additionally, people from the Central region were the least likely to have no opinion on this matter, whereas interviewees from the North-Western region were the least likely to disagree with the statement that such relationships are formed.

However, it is worth pointing out that none of the relations were strong despite their statistical significance. Strength of effects in statistically significant relations between variables varied between 0,109 in the weakest relation, which was between Macro-region and statement that “Ukrainians destabilise the Polish economy” and 0,169 in the strongest relation education and statement that “Ukrainians take jobs away from Poles” (Table 2). These results show, that none of the relations was particularly strong, as V-Cramer value below 0,3 means weak strength of effect.

### 2. Characteristics of stay

**Fig 4:**
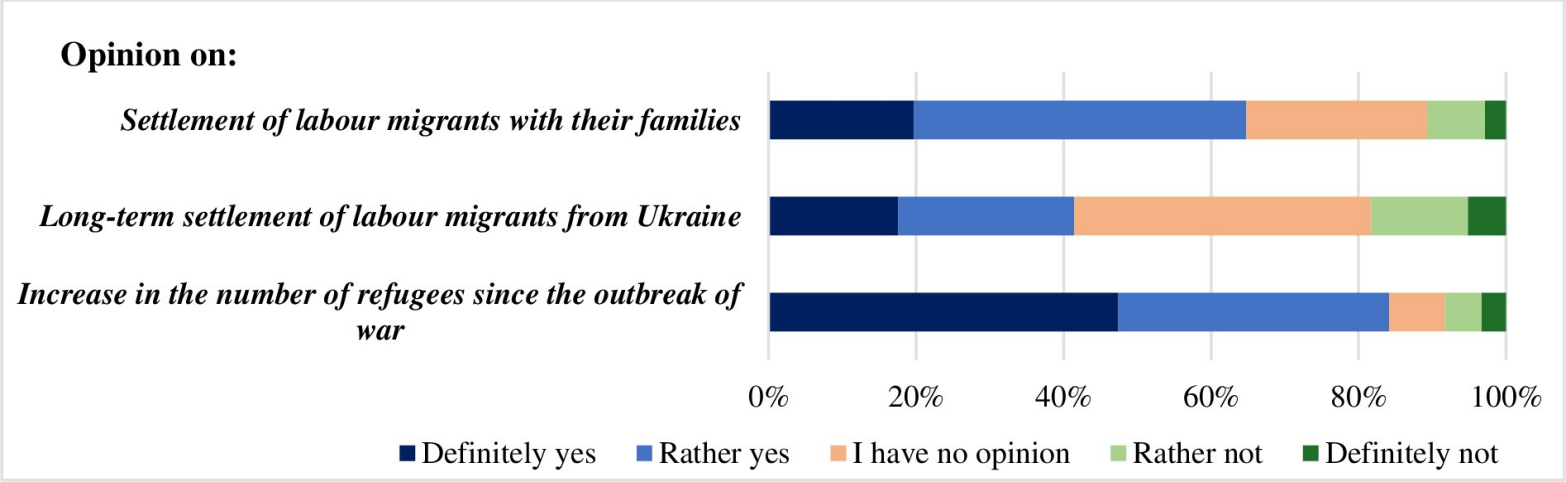
Opinions of the respondents on the characteristics of the stay of Ukrainian migrants in Poland.

Most of the respondents (84.1%) noticed an increase in the influx of migrants from Ukraine after the Russian invasion (Fig 4). Notably, 64.7% of them emphasized that the Ukrainians were bringing their families along, which may be beneficial to the future of demographics in Poland. It may also be linked to the fact that over 41.5% of the respondents believed that the migrants would stay in Poland for longer.

Nevertheless, it is worth noting that all of the relationships should be described as weak despite their statistical significance. The strength of effects in relationships assessed as statistically significant, with p-values, ranged from 0.141 in the weakest relationship (between sex and ‘Settlement of labor migrants with their families’) to 0.210 in the strongest relationship (between education and ‘Increase in the number of refugees since the invasion’) (Table 3). Looking at this result, one can state that none of the relationships were particularly strong, as all Cramér’s V values below 0.3 indicate a weak strength of effect.

**Table 3:** Results of the test of independence of respondents’ opinions about the characteristics of the stay of migrants from Ukraine in Poland by sex, age, education, and residence.

Specification								
	Sex		Age		Education		Macro-region	
	*p-value*	Cramér’s V	*p-value*	Cramér’s V	*p-value*	Cramér’s V	*p-value*	Cramér’s V
*Increase in the number of refugees since the invasion*								
	-		-		***	0.210	-	
*Long-term settlement of labour migrants from Ukraine*								
	-		-		-		-	
*Settlement of labour migrants with their families*								
	**	0,141	-		-		-	

p value

* *p* < 0.05

** p < 0.01

*** p < 0.001

‘-’ p > 0.05

**Fig 5:**
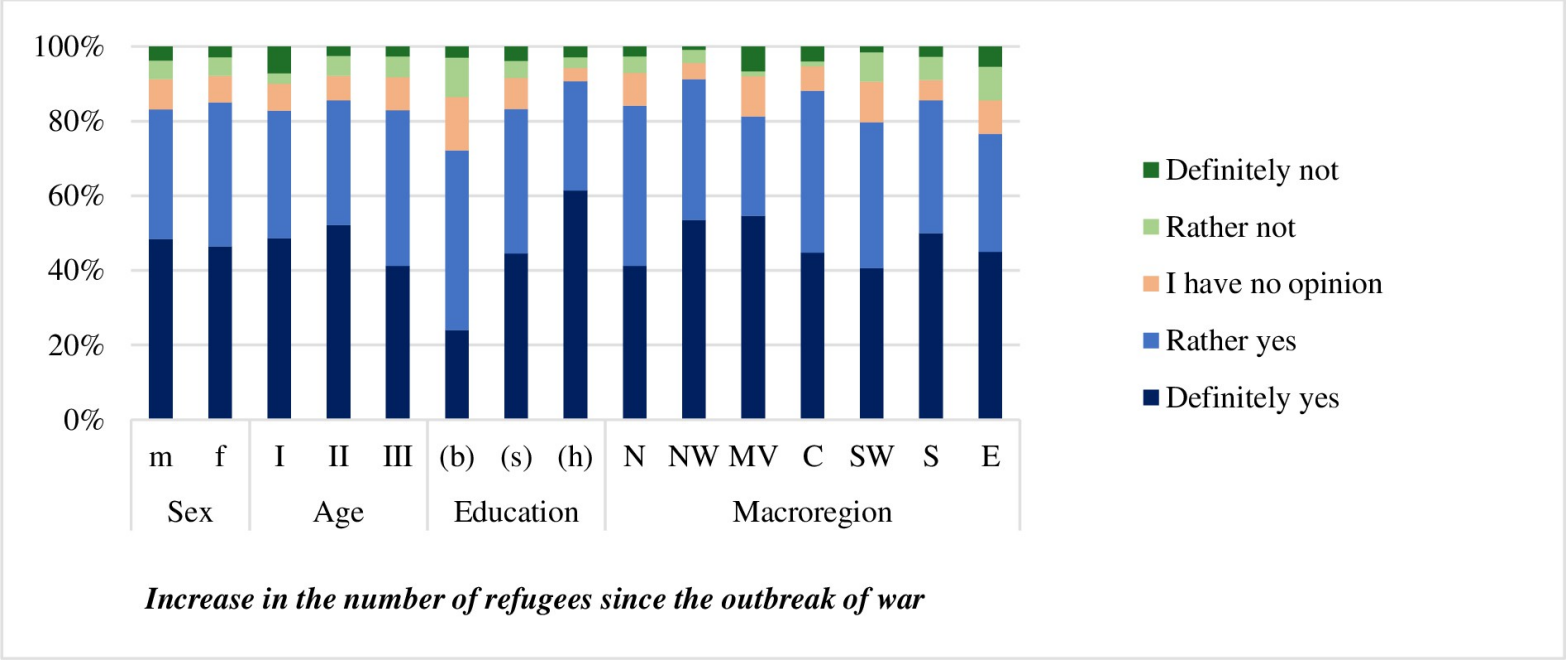
Opinions of the respondents about the characteristics of the stay of Ukrainian migrants in Poland by sex, age, education, and macro-region. Symbols as per Table 1.

The strongest differentiating factor for the answers given to these questions was the educational background. Higher education entailed affirmative answers to question about increased number of refugees since the outbreak of war, where people with higher education were much more prone to notice higher influx of migrants (Fig 5). It might be related to the fact, that they are more likely to be engaged in organisations working with migrants from Ukraine.

There were no factors significantly differentiating the respondents regarding the length of stay of the migrants (Table 3). Sex affected the answer regarding the settlement of migrants with their families (Table 3). Women were more likely to state that migrants settle with their families, whereas men twice almost twice as often had no opinion in this matter. Age and macro-region significantly affected none of the answers tested in this group.

### 3. Institutional support for Ukrainian migrants

Nearly half of the respondents (49%) agreed that the support system for Ukrainian migrants was a burden on municipal budgets (Fig 5). This opinion was expressed more often by women (55%) than men (43.7%). As much as 63% of the respondents in the young age group (18–34) agreed with that statement. The older age group (35–54) agreed to a lesser extent (49%), while the oldest group agreed in 45%. The educational background had a statistically significant impact on the selected answer. More than half of people with basic and secondary education (54%) believed migrant support to be a burden on municipal budgets. This statement resonated most with residents of South-West.

**Fig 6:**
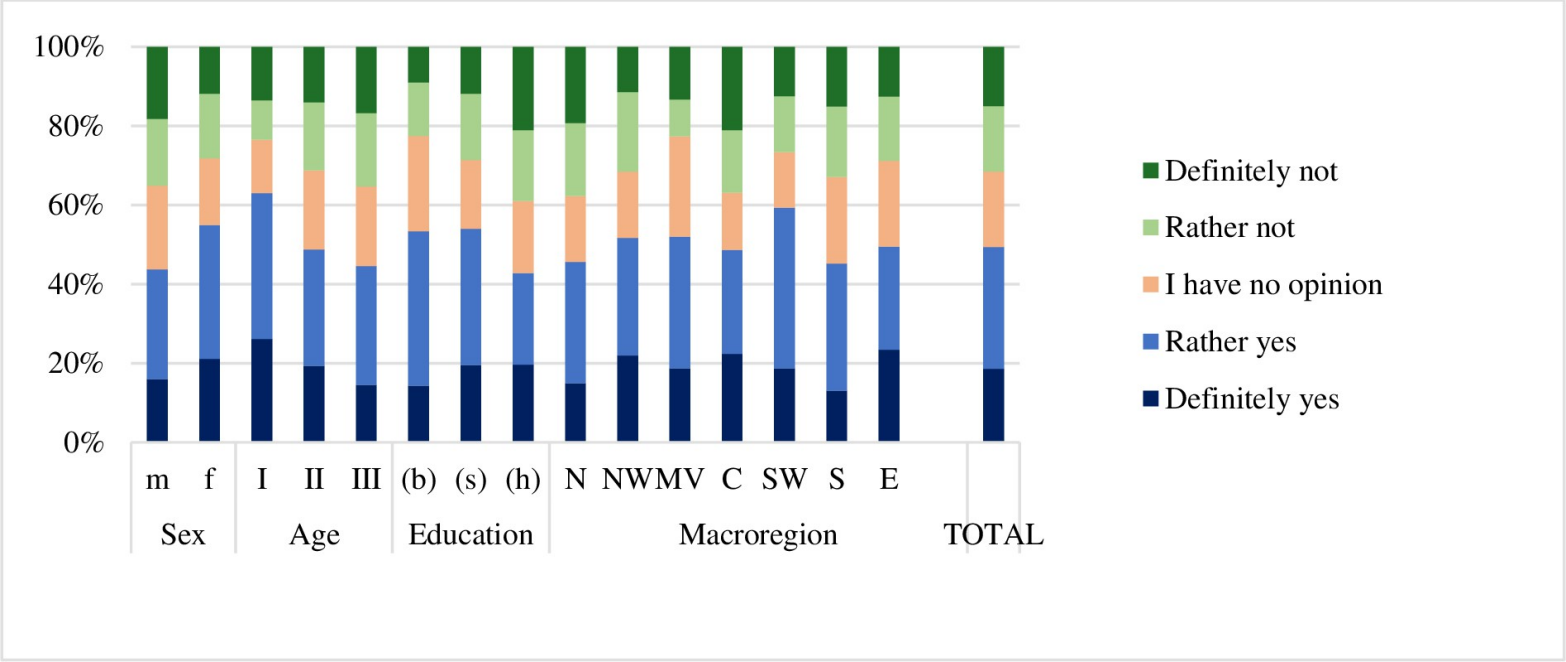
Opinions of the respondents on whether the support for Ukrainians burdened the municipal budget in Poland by sex, age, education, macro-region, and for the entire sample with independence test results.

Response ‘I don’t know’ concerning the support measures used by migrants from Ukraine who stayed in the respondent’s municipality was given by 39.4% of the respondents (Fig 6). The percentage varied across the macro-regions from 30.7% (Mazowieckie) to 43.9% (North).

The respondents were the most aware of support benefits to cover maintenance, in particular food, clothing, footwear, personal hygiene products, and lodgings fees. Awareness of health insurance contributions covered by the state is slightly smaller.

**Fig 7:**
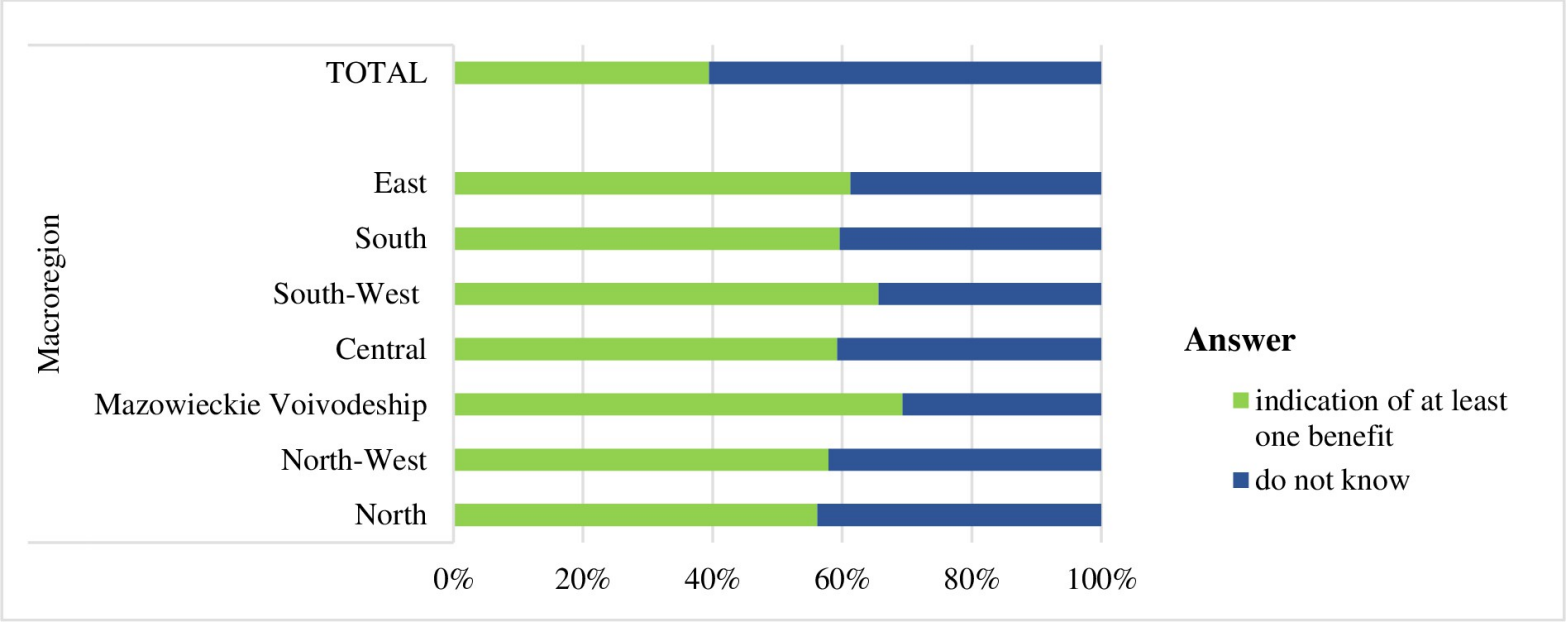
Structure of responses regarding the respondents’ awareness of support (welfare) means used by migrants from Ukraine who stayed in the respondent’s municipality by macro-region.

**Fig 8:**
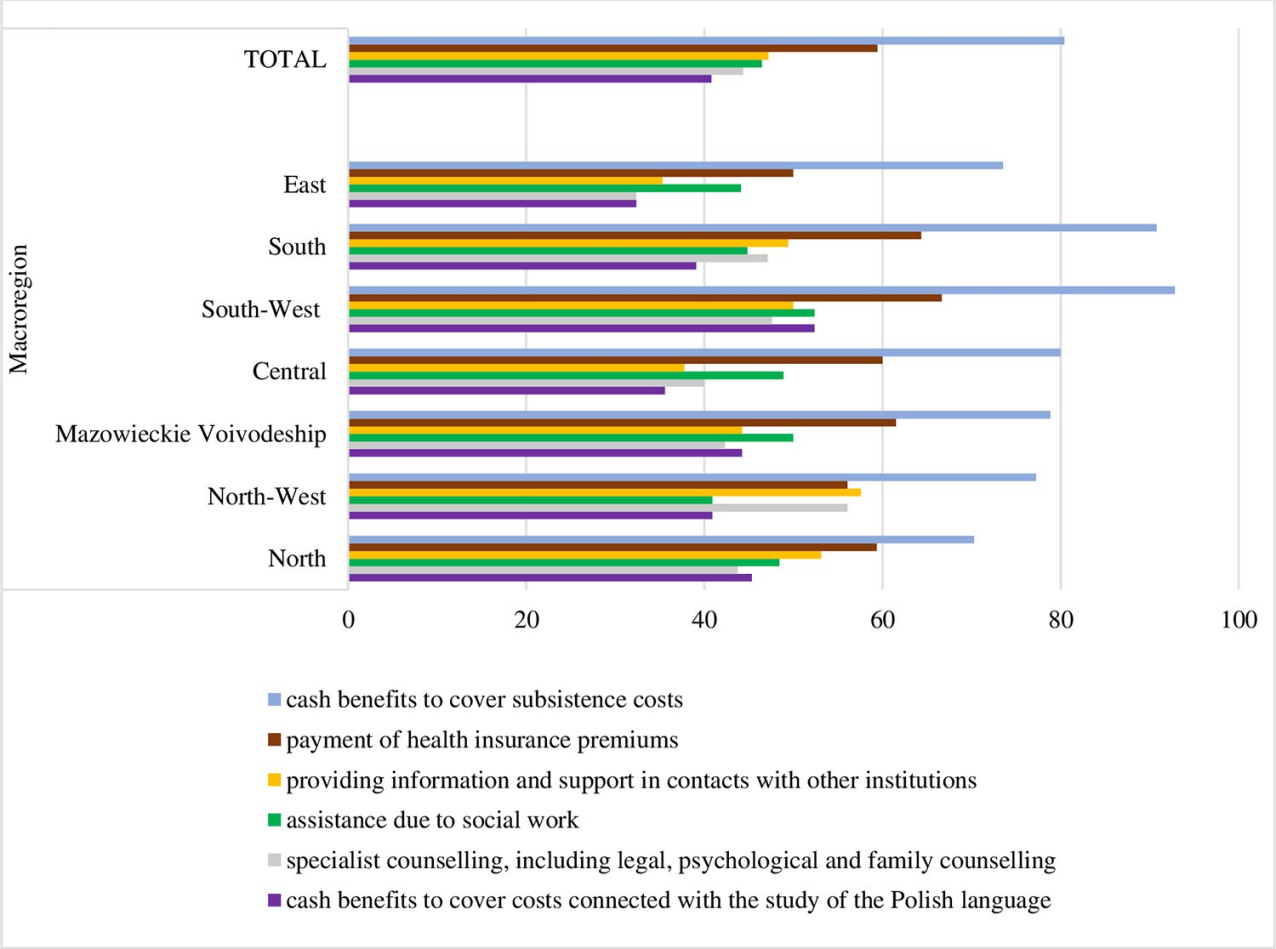
Indications of support (welfare) means used by migrants from Ukraine who stayed in the respondent’s municipality [percentage of declarations of awareness of support means].

The South-West and Mazowieckie Voivodeship macro-regions exhibited the highest level of awareness concerning support benefits for migrants from Ukraine (Figs 7 and 8). The least knowledgeable in this regard were residents of North and East.

## Discussion

It has been over a year since the largest exodus of Ukrainians fleeing the war in their country in the history of Poland and Europe. From 1.2 to 1.3 million of them settled in Poland in addition to those who lived there before. Researchers from the Faculty of Political Science and International Studies, University of Warsaw and University of Economics and Human Sciences in Warsaw probed the public perception of Ukrainian refugees for the second time in January 2023. The survey results show that the positive attitude of Poles towards Ukrainians and support for them—mainly military—have remained virtually the same since the invasion. Nevertheless, a quarter of the respondents gave the affirmative answer to an additional question: ‘Has your attitude towards migrants from Ukraine changed over the last six months from June 2022?’ Moreover, 68% of them declared the change was towards a negative perception, which meant a worse attitude. As the report reads, ‘the following are listed among the potential threats to Poland posed by migrants from Ukraine:

- an adverse impact on the Polish economy / state budget / inflation;
- an adverse impact on the labour market;
- crime’.

The impact of Ukrainian nationalism declined compared to April 2022, but the respondents mentioned entitlement mentality and different culture of Ukrainians more often [20].

According to Łodziński and Szonert [21], the migration policy is becoming a policy without politics: coherent efforts in various aspects of migration, such as the labour market, policy towards the international community of Poles, protection of borders, and refugee policy with no broader and official discussion concerning its long-term goals. In this policy without politics, priorities tend to congregate around the needs of the labour market, demographics, and migration, considering the actual political context instead of ideological and strategic presumptions.

The impact of stereotypes on the way to integration is an important social aspect [22, 23]. The strength of negative stereotypes and negative narration of media [24] in both cultures (Polish and Ukrainian) stemming from past armed and social conflicts between the nations will most probably dwindle gradually as their coexistence continues [25]. Immigrants and refugees face the most significant challenges with transferring their skills and adapting to the host country’s labour market [26]. Economic immigrants, who base their decisions on how easily their human capital and other resources can be transferred to host countries, tend to be more favourably selected to enter the labour market [27]. Migrants tend to move to countries where their skills are relatively scarce, filling in gaps in the natives’ set of skills [28].

The Ukrainians’ continuing desire for their own homeland should be considered. So, finding one’s own place among the Polish community is a big challenge. More civic-oriented identity becomes the predominant approach to nation-building in Ukraine, citizenship will be a central part of the process [29–33].

As regards assistance to Ukrainians, the public is divided along another line as well. Nearly half of the respondents (48%) believed Poland should provide more support, but more than a third were against it. Some called for curbing welfare. The researchers with the University of Warsaw pointed out another trend that had been there all along and has deteriorated slightly recently. Approval for the Act on aid to Ukrainian nationals (which grants them benefits equal to those for EU citizens) has slightly declined by 4 pp. since April 2022. Although most Poles are in favour of aid to Ukrainians in the form of a single payment of PLN 300 (49%), access to a free healthcare system (62%), and admission of children of Ukrainian refugees to Polish schools (87%), there is no broad approval of family and parent benefits, like PLN 500 monthly per child or social welfare benefits. Five per cent more people perceive it negatively than in 2022. Up to 16 pp. more (36% today) of the respondents were not in favour of state-funded lodgings for Ukrainians. In February, the Polish government introduced partial payment for refugees who spent more than 120 days in Poland. The public is annoyed by the more widespread foreign language, mostly Russian, used by the refugees. It is perceived as a ‘language of the enemy’, and Poles become disoriented as to who they are [20].

Other important efforts include governmental, non-governmental, and business initiatives to help the refugees find employment, such as Polish language courses, vocational training (including IT), job board applications and websites for Ukrainians, including governmental pracawpolsce.gov.pl opened in summer 2022 [34].

Selected cash benefits available to Ukrainians after the invasion:

- PLN 300, a one-time livelihood benefit;
- PLN 500, a child benefit under the Rodzina 500+ scheme;
- family care capital for every child aged 12 to 35 months;
- PLN 95 to PLN 135 a month of a family benefit;
- PLN 1,000, parent benefit;
- PLN 215.84, nursing benefit;
- nursing pay;
- PLN 1,000 for childbirth;
- care benefit;
- up to PLN 400, subsidy for daycare centre, children’s club, or daycare provider;
- PLN 300, Dobry Start scheme for schoolchildren;
- PLN 90 to PLN 110, allowance for education and rehabilitation of a child with disabilities;
- rent allowance;
- welfare ticket;
- meals for children and students from Ukraine;
- reimbursable medicines;
- PLN 500, Health4Ukraine medicines allowance;
- benefits granted under specific conditions and for a specific time under the Act on aid to Ukrainian nationals related to the war in Ukraine of 12 March 2022.

The Polish government has provided substantial aid to the refugees, just like local governments, grassroots organizations, and the people. According to research by the Polish Economic Institute, the total (annual) public assistance and private aid (estimated for the first three months after the invasion) provided to the refugees is around PLN 25.4 billion, or 0.97 per cent of the Polish GDP for 2021 [7].

An additional mobility barrier concerns the large heterogeneity in social insurance rights across European countries. These rights, including old-age pensions, unemployment payments, and government-financed healthcare services, are determined at the national level, and programs differ strongly across countries [35].

Economic aspects of migration were also investigated in a survey among 808 employers from Małopolskie and Podkarpackie Voivodeships who employed migrants from Ukraine and had a business in one of the voivodeships between 2014 and 2018. The most represented sectors were construction (31.7%) and industry (15,8%). Employers representing services (15.5%) and trade (13.7%) were also rather numerous. The share of the other sectors did not exceed 10%. The survey results show that the main reason behind hiring Ukrainian employees is cost reduction through lower remuneration compared to Polish workers. This argument was provided by 57.3% of the surveyed employees. The result is not significantly varied across the population. The second most popular reason was permanent issues with finding employees with vocational profiles for specific existing vacancies in the local labour market. This reason was provided by 46.3% of the employers. The third reason was better efficiency, quality of work, availability, or work discipline of Ukrainian workers compared to their Polish colleagues. This factor was provided by 40.2% of the employers [36].

But, there is the distinct local character of the demands for foreign workers in the Mazovian region, and specifically in the direct surroundings of Warsaw. This is a region with relatively strong and market-oriented horticulture sector that has employed temporary migrant workers on a massive scale for over a decade [37].

Results of a 2022 survey on 1481 refugees from Ukraine who came to Opolskie Voivodeship (south-western Poland) revealed their relatively high competencies. An overwhelming majority of the refugees (62.7%) had both qualifications and professional experience. Moreover, nearly half of them (48%) had a university degree; they were mostly economists (18.9%) psychologists and educators (14.9%), engineers and technicians (10.8%), and physicians (8.4%) followed by representatives of humanities and law graduates (7.4%), personal services (7.5%), and management (6.1%). Trade, production, and transport professionals constituted a much smaller group: 4.2%, 4.6%, and 1.4%, respectively [38].

According to the portal Rynek Pracy [39], the number of foreign nationals, including Ukrainians, who get employment and social insurance is growing. In the first quarter of 2019, the Social Insurance Institution had 610,000 foreign nationals registered, including 455,000 Ukrainians. The number grew to 1,057,000 foreign nationals in the first quarter of 2023 with 738,000 Ukrainians. The number of Ukrainians with Polish social insurance grew significantly from 2019 to 2023.

In mid-February 2023, the EWL Migration Platform employment agency [40], Centre for East European Studies of the University of Warsaw [41], and the EWL Foundation for Support of Migrants on the Labour Market conducted a survey on 400 adult Ukrainians from across Poland. It demonstrated that 82% of adult refugees from Ukraine found employment in Poland, reaching 84% in the working-age group. The largest share (34%) started working within one to three months after arrival. This high level of employment within the first months of their stay reflects the determination of the refugees to find a job, the openness of Polish businesses to new employers, and the needs of the Polish market and economy.

Migrants from Ukraine fuelled a short-term spike in retail and private consumption in 2022, especially in Poland and Estonia. Moreover, as they grow more integrated, they will join the workforces of the host countries and improve production output in the medium and long term. Ukrainian migrants abroad drive private consumption in host countries, contributing to economic growth. According to National Bank of Ukraine data, Ukrainians abroad spent two billion dollars a month in 2022, over three times more than in the previous year. Estimates by the International Monetary Fund and study by the United Nations, controlled for all other conditions, forecast the contribution of Ukrainian migrants to improve production compared to a base scenario without migration by 2.2 per cent to 2.3 per cent in Estonia, Poland, and Czechia, and by 0.6 per cent to 0.65 per cent in Germany in 2026. Integration of migrants from Ukraine will affect labour markets in host countries [42].

The European Central Bank, in particular, expects the share of working-age Ukrainian migrants to reach 25% to 55% of the workforce in the Eurozone in the mid-term. Although in the short term, migrants pose additional challenges to the state budget, they will probably be beneficial to the finances and economy of host countries if they remain in the countries for more than several years and are active in the labour market [43].

Bird and Amaglobeli [44] estimated the short-term fiscal impact of migrants from Ukraine on the economies of EU states to reach EUR 30–37 billion, which is 0.19–0.23% of the EU’s GDP. Ukraine’s neighbours and Baltic states will spend the largest sums. According to the European Investment Bank [45], Latvia could spend 9% of its GDP on the adaptation of migrants, Estonia over 7%, and Hungary, Poland, and Czechia, 4–6%. Estimates by the International Monetary Fund expect the long-term net fiscal effect to be beneficial to Europe because Ukrainians actively integrate with the European labour market. Taxes paid by Ukrainian migrants in Poland are a particularly good example of their integration with the labour market.

Regarding Ukrainian nationals in the Polish labour market, 78% of respondents in a 2020 survey among 502 temporary workers from Ukraine working in Poland through OTTO Work Force companies were satisfied with working in Poland. It is 6 pp. more than in the previous year when the workers were more critical. The value for 2022 was virtually the same as for 2020 (79% and 78%, respectively). The percentage was the highest in 2017 (94%) which may be because June 2017 was the first time Ukrainian nationals could legally stay in Poland under the visa-free regime [46].

The Centre of Migration Research of the University of Warsaw estimated that Ukrainians paid PLN 10 billion in taxes in Poland (about USD 2.4 billion) [47].

In summary, the rebuilding of Ukraine after the war will probably offer an opportunity for Polish business [48–51]. The networks of enterprises that are being established today will be more effective in the new socioeconomic environment [52–54]. Demographics are an important determinant of the contribution of Ukrainian migrants to the economic growth of Poland [55, 56]. Refugees coming to Poland are mainly women with children [57]. Their permanent settlement in Poland will improve demographic indicators (more children), which is of value in light of the ageing population. Migrants’ labour market integration is a topic of increasing public concern, particularly in the light of the recent refugee crisis faced by mainland Europe. However, non-economic migrants should not be seen as an inevitable burden but as further investments in the host country whose relevant skills can serve to close existing labour market gaps and improve their integration substantially [58]. Public opinion toward migrants is not positive and explicitly negative during economic downturns. However economic benefits of labour immigration overweight disadvantages. As the Brexit poses the first precedent since WWII when the economic and social ties in Europe loose [59].

The labour market situation of migrants from Ukraine is changing month by month. It is shaped by several factors: 1. Pre-war migrants active in the agricultural and horticulture industries (the primary targets of seasonal migration in rural and town areas in Poland) are mainly men. Some of them, especially younger ones, no longer migrate due to the war. 2. Before the war, work in Polish agricultural and horticulture industries had been more financially beneficial than during the war, as the geographic reach of migration is growing (towards Western Europe).3. The perception of Ukrainians in Poland is changing because of the war, aid to the migrants, and the growing burden of cultural differences. Therefore, future research should focus on seasonal migrations in the context of broader demographic and social characteristics of the migrants, the profitability of seasonal work in Poland for Ukrainians, and relationships between Poles and Ukrainians in the context of their stay in Poland.

## Conclusions

Most foreign nationals in the Polish labour market in 2021 were Ukrainians. The positive trend started in 2017 when they could enter Poland without a visa for the first time. The number of Ukrainian workers in the Polish labour market declined compared to other foreign nationals from 2021 to 2022. The change was not due to a smaller number of Ukrainian employees but the Act, which allows for employment without a permit. The number of permits issued by individual macro-regions varied from 2017 to 2022. The leading macro-region changed from Mazowieckie Voivodeship to North-West (closer to the German border).

The main research objective is to diagnose the conditions of seasonal labour migration, considering sex, age, education, and region of residence of the respondents. The respondents perceived the migration of Ukrainian nationals to Poland mostly positively, especially regarding seasonal work. The youngest respondents and those from the Central and East macro-regions were the most sceptical about the positive impact of Ukrainian migration on the Polish economy. Those with higher education, living in the North, and from Mazowieckie Voivodeship were more optimistic. They also emphasized that the Ukrainians performed work at variance with their qualifications. Only every fifth participant agreed that migrants took away jobs from Poles. Most of the respondents pointed out that small business owners benefited from employing Ukrainians.

A specific objective includes characteristic of stay. The overwhelming majority of the respondents noted an increase in migration from Ukraine after the full-scale invasion and that entire families of Ukrainians were coming to Poland. Less than half of the interviewees believed the migrants would stay in Poland for longer. People with university degrees pointed out the increased number of migrants following the full-scale invasion. It may be because they are more often involved in the efforts of organisations that help migrants from Ukraine. Women more often stated that the migrants settled together with families.

A last specific objective concerns an institutional support for Ukrainian migrants. Nearly half of them agreed that the support system for Ukrainian migrants was a burden on municipal budgets. Most in this group were women and people aged 18–34. At the same time, 40% of the survey population did not know what support means were used by migrants from Ukraine. The respondents were the most aware of support to cover maintenance, in particular food, clothing, footwear, personal hygiene products, and lodgings fees. Participants from the Central and East macro-regions were not inclined to perceive Ukrainians as a growth opportunity for the Polish economy. Residents of East and North had poor awareness of welfare benefits migrants from Ukraine were eligible for.

The study is limited by the systemic solutions presented in the article, which fail to include actions dedicated to the specific characteristics of regions in Poland. The results demonstrate varied acceptance for migrants depending on the residence, sex, and age of the respondents. The diagnosis of the labour market and work conditions of seasonal migrants was conducted considering the historical memory of Poles and stereotypes affecting the opinions on support for Ukrainians. This conclusion and others offered here may be useful when defining objectives for a regional policy regarding seasonal immigrant labour. Researchers investigating the problem of immigrants in the seasonal labour market face the challenge of factoring in applicable labour policies, work conditions, remuneration, and other quantities considering employers and migrant employees. Such a broader research perspective can juxtapose the results with the effectiveness of governmental programmes aimed at supporting seasonal migrant workers. The public opinion survey presented in the article has to be expanded with future research considering respondents’ experience and professional contacts with Ukrainians and how they are supported in the labour market.

## Supporting information

S1 Checklist Human participants research checklist. (PDF)
